# Rb Suppresses Collective Invasion, Circulation and Metastasis of Breast Cancer Cells in CD44-Dependent Manner

**DOI:** 10.1371/journal.pone.0080590

**Published:** 2013-12-04

**Authors:** Kui-Jin Kim, Alzbeta Godarova, Kari Seedle, Min-Ho Kim, Tan A. Ince, Susanne I. Wells, James J. Driscoll, Samuel Godar

**Affiliations:** 1 Department of Cancer Biology, Vontz Center for Molecular Studies, University of Cincinnati College of Medicine, Cincinnati, Ohio, United States of America; 2 Biomedical Research Center, Ulsan University Hospital, Ulsan, Republic of Korea; 3 Department of Pathology, Braman Family Breast Cancer Institute and Interdisciplinary Stem Cell Institute, University of Miami Miller School of Medicine, Miami, Florida, United States of America; 4 Division of Oncology, Cancer and Blood Diseases Institute, Cincinnati Children's Hospital Medical Center, Cincinnati, Ohio, United States of America; 5 Division of Hematology and Oncology, University of Cincinnati College of Medicine, Cincinnati, Ohio, United States of America; University of Texas MD Anderson Cancer Center, United States of America

## Abstract

Basal-like breast carcinomas (BLCs) present with extratumoral lymphovascular invasion, are highly metastatic, presumably through a hematogenous route, have augmented expression of CD44 oncoprotein and relatively low levels of retinoblastoma (Rb) tumor suppressor. However, the causal relation among these features is not clear. Here, we show that Rb acts as a key suppressor of multiple stages of metastatic progression. Firstly, Rb suppresses collective cell migration (CCM) and CD44-dependent formation of F-actin positive protrusions in vitro and cell-cluster based lymphovascular invasion in vivo. Secondly, Rb inhibits the release of single cancer cells and cell clusters into the hematogenous circulation and subsequent metastatic growth in lungs. Finally, CD44 expression is required for collective motility and all subsequent stages of metastatic progression initiated by loss of Rb function. Altogether, our results suggest that Rb/CD44 pathway is a crucial regulator of CCM and metastatic progression of BLCs and a promising target for anti-BLCs therapy.

## Introduction

Migration of cancer cells is an initial step in multistep process of metastasis during which malignant cells spread from the primary tumor to distant organs [1]. Depending on the cell type and tissue environment, cells use two major modes of migration: individual, single cell migration (SCM), when cell-cell junctions are absent, and multi-cellular, collective cell migration (CCM), when cell-cell adhesions are retained [2]. CCM is particularly important during embryogenesis and postnatal development, when it drives the formation of various tissues. A prime example of developmental CCM relevant to breast tumorigenesis is the expansion of terminal end buds that compels expansion of mammary epithelium and requires cooperation of cells with both luminal and basal phenotypes [3]. Invasive breast carcinoma cells may hijack this or similar mechanisms and reactivate CCM in response to suitable oncogenic stimuli.

The most aggressive subset of breast carcinomas is basal-like carcinomas of the breast (BLC). BLCs have poor prognosis, exhibit resistance to anti-estrogen therapy, and lack any known clinically-proven therapeutic target such as Her-2. Their major histopathological features, in addition to lymphovascular invasion, are extensive necrosis in the primary tumor and metastatic spread to the lungs and brain [4]. However, the molecular mechanism of lymphovascular invasion, its potential role in dissemination of circulating cancer cells (CCC) and the subsequent colonization of the target organ are poorly understood.

Recent evidence suggests that two important molecular characteristics of BLCs are decreased expression of Rb tumor suppressor [5], and elevated expression of CD44 [6], a marker of breast cancer stem cells [7]. Rb initiates and maintains cell cycle arrest, modulates apoptosis, and is essential for early embryonic development but is dispensable for the survival of mammary epithelium [8], [9]. Inactivation of Rb in mouse mammary epithelium induces aggressive and metastatic mammary tumors with features of the basal stem cell phenotype [10], [11], indicating that Rb is an important negative regulator of cell growth as well as primary and metastatic mammary tumor growth with basal differentiation. However, the potential role of Rb in suppressing malignant characteristics of BLCs, e.g., lymphovascular invasion, hematogenous metastatic spread, or upregulation of CD44 protein expression, is unknown.

CD44, an alternatively spliced transmembrane protein, functions as a receptor for hyaluronan and as a co-receptor for multiple receptor kinases that have been linked to breast cancer [12]. We have previously shown that the tumor suppressor, p53, inhibits CD44 expression and prevents it from compromising growth-inhibitory, pro-apoptotic, and tumor suppressor functions of p53. We also found that CD44 expression is essential for maintaining the cancer stem cell phenotype and for primary tumor growth of mammary cells with mixed basal/luminal characteristics and inactivated p53 and Rb function [13], [14].

The aforementioned evidence prompted us to hypothesize that Rb acts as the key suppressor of metastatic progression at multiple levels. Indeed, suppression of Rb expression resulted in metastatic stimulus that initiated CCM, lymphovascular invasion, cell-cluster-based dissemination of metastatic cells from primary tumors, and consequent metastatic growth. We also provide evidence that upregulation of CD44 levels caused by loss of Rb is essential for all these mentioned stages of metastasis revealing crucial role of Rb/CD44 axis in etiology of metastasis.

## Results

### Rb suppression stimulates CCM in vitro and lymphovascular invasion, lung metastasis and CD44 expression in vivo

**Figure 1:**
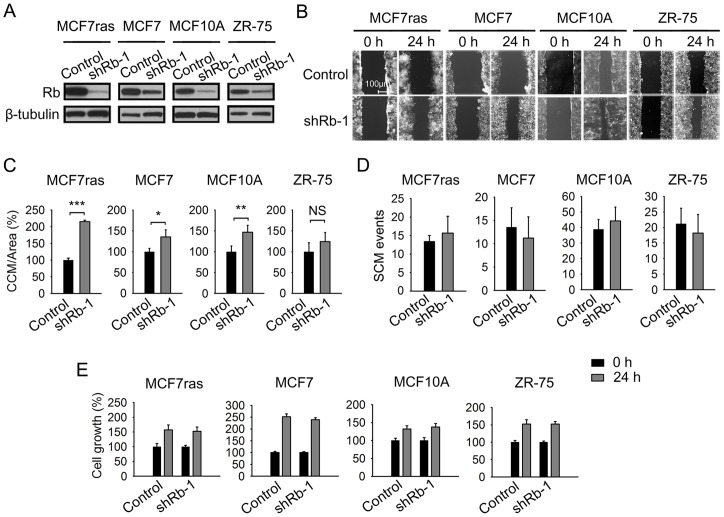
Suppression of Rb expression in human breast cancer cell lines stimulates collective cell migration. (A) Breast cancer cell lines were infected with a lentiviral construct expressing puromycin resistance gene and Rb-1 or control shRNA. Rb-knockdown efficiency in breast cancer cell lines was determined by a western blot. (B) Collective cell migration (CCM) assays for the indicated breast cancer cell lines derivatives. Scale bar, 100 μm. (C) Quantification of CCM for indicated breast cancer cell lines. CCM was quantified as an area covered between initial point and 24 hour point, and expressed as a percentage relative to the control. The experiment was performed three times in triplicate, data are presented as mean ± SD; equal variance Student's t-test, * p<0.05, ** p<0.01, *** p<0.001; NS indicates difference that was not significant. (D) Quantification of single cell migration (SCM) assays. Cells derived from indicated breast cancer cell lines were allowed to migrate overnight. The experiment was performed three times in triplicate. Data are presented as mean ± SD. (E) Breast cancer cell lines derivatives were analyzed 24 hours after seeding by MTT assay. Data from a representative experiment (n = 5) performed in triplicate are expressed as amount of metabolized MTT measured by absorbance normalized to the absorbance of control shRNA and presented as mean ± SD.

**Figure 2:**
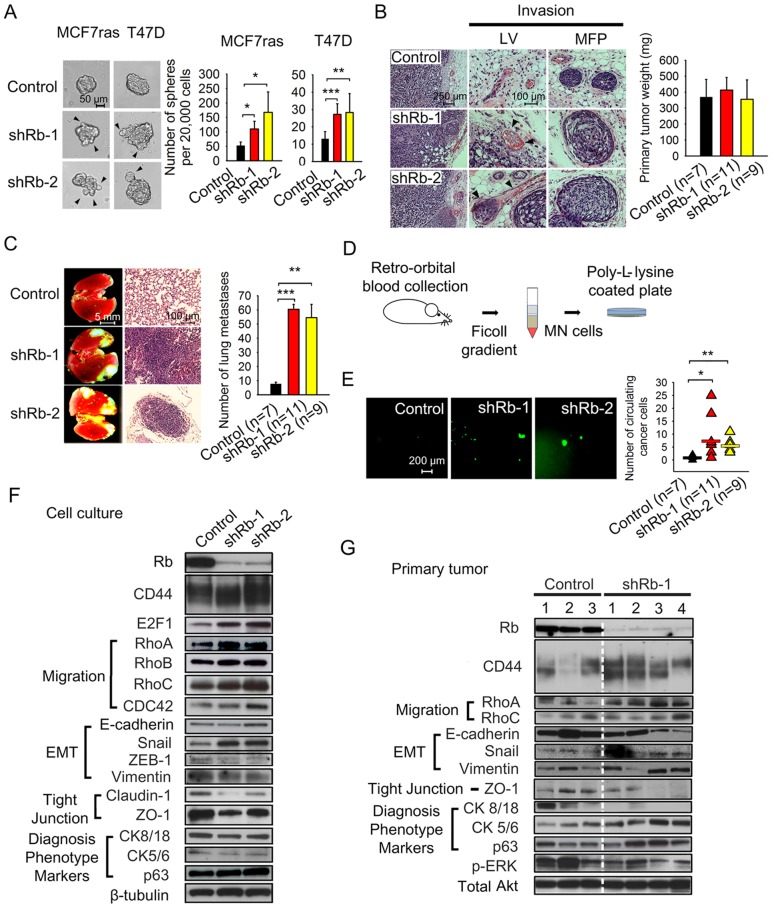
Loss of Rb expression promotes metastatic behavior in human breast cancer cell lines. (A) Phase contrast images and quantification of mammosphere-forming potency of MCF7ras and T47D cells with Rb knockdown. Arrows indicate protrusions formed by invading cells and cell clusters. Scale bar, 50 μm; unequal variance Student's t-test, * p<0.05, ** p<0.01, *** p<0.001. (B) Morphology of primary tumors and lymphovascular (LV) or mammary fat pad (MFP) invasion. MCF7ras cells stably expressing shRNA against Rb or scrambled sequence were injected into the MFP of NOD/SCID mice. Primary tumors and other tissues were harvested after 8 weeks and H&E stained. Bar graph depicts primary tumor weight in each group expressed as mean ± SD where *n* is the number of animals in each group. Arrows indicate sites of lymphovascular invasion in Rb knockdown experiment. Scale bar, 50 μm (left panel) and 25 μm (middle panel). (C) Fluorescent and H&E stained images of metastatic tumor growth in lungs. Metastases containing EGFP-expressing Rb knockdown or control cells originated from orthotopic site. Images from *n* number of animals were quantified using ImageJ software (NIH). Data are presented as mean ± SD. Scale bar, 5 mm or 100 μm; equal variance Student's t-test, ** p<0.01, *** p<0.001. (D) Scheme showing isolation of circulating cancer cells (CCC) from mice bearing primary tumors. CCC were co-separated with the mononuclear fraction of blood and adhered to Poly-L-lysine-coated plates. (E) Fluorescent images and quantification of isolated EGFP-expressing circulating cancer cells and cell clusters. Scale bar, 200 μm; unequal variance Student's t-test, * p<0.05, ** p<0.01. (F) Western blot analysis of cell lysates from in vitro cultures of MCF7ras cells expressing Rb or control shRNA. β-tubulin staining was used as a loading control. (G) Western blot analysis of lysates from primary tumors. Total Akt was used as a loading control.

To test our hypothesis that loss of Rb function can initiate CCM, we examined the effect of Rb knockdown (Figure 1A) on collective and single cell-based migration in vitro. We analyzed CCM using a scratch assay and SCM by Boyden chamber type assay. Rb knockdown stimulated substantial CCM but not SCM in MCF7ras cells (Figures 1B–1D) and comparable effect was seen with another independent shRNA (Figures S1A and S1B). Similar results were obtained with MCF7 cell line as well as with immortalized but not tumorigenic MCF10A cells (Figures 1B–1D). Tendency of ZR-75 cells with Rb knockdown to migrate collectively did not reach statistical significance (Figure 1C). Cell growth for all cell lines tested was independent of Rb expression (Figures 1E and S1C). These results indicate that suppression of Rb expression preferentially stimulates CCM over SCM in vitro. The proclivity to collective invasion was also highlighted by the irregular budding patterns of spheres originating from Rb knockdown cells compared to the relatively smooth surface of control spheres in MCF7ras and T47D cells (Figures 2A and S1D). Rb knockdowns also had higher sphere-forming ability (Figure 2A).

To explore the impact of Rb knockdown on cell invasion and metastasis, we chose a well-established mammary carcinoma model: MCF7ras cells that can form tumors independent of estrogen signaling [15]. To address the role of Rb in metastatic progression we infected MCF7ras cells with recombinant lentiviruses that stably express either enhanced green fluorescent protein (EGFP) or antibiotic resistance gene, and shRNAs to Rb. We then implanted MCF7ras cells with Rb knockdown into mouse mammary fat pads (MFPs). Rb knockdown had no significant effect on primary tumor growth (Figure 2B, right panel). However, cells with Rb knockdown exhibited substantial extratumoral lymphovascular (LV) invasion (Figure 2B, left panel) and massive metastatic growth in lungs (Figure 2C). Diminished Rb also led to cell cluster-based invasion into MFP that could not be clearly identified as lymphovascular invasion, but could be of lymphovascular, ductal or other origin. This type of invasion we termed MFP invasion. Postmortem analysis of blood revealed increased frequency of EGFP-positive CCC with larger, possibly multicellular clusters in hematogenous circulation (Figures 2D and 2E) in animals implanted with Rb knockdown cells. The EGFP-positivity of the cancer cells in the circulation suggested that these cells had intact membranes and were most likely alive. Altogether, inactivation of Rb stimulated lymphovascular/MFP invasion and hematogenous metastatic spread, both typical features of BLCs.

We proceeded to dissect the molecular mechanism of CCM in cells lacking functional Rb by analyzing the expression of proteins known to be involved in cell motility as well as expression of epithelial and differentiation markers (Figure 2F). We found consistent upregulation of Rho A and Rho C, proteins known to stimulate cell motility [16], in Rb knockdowns. Because increased motility is often associated with epithelial to mesenchymal transition (EMT) [17] we decided to analyze expression of EMT markers in Rb knockdown cells. While expression of the epithelial marker E-cadherin and the mesenchymal marker vimentin was not significantly altered in MCF7ras cells, Snail transcription factor was upregulated in cells with Rb knockdown. This is consistent with downregulation of tight junction proteins claudin-1 and ZO-1 that were described to be suppressed by Snail independently of E-cadherin [18]. Claudin-1 expression was inhibited in other breast cancer cell lines as well (Figure S2A) indicating that the downregulation of tight junctions represents a possible molecular mechanism that facilitates CCM in breast cancer. Expression patterns of EMT markers also suggested that Rb knockdown cells undergo a partial EMT that may not affect adherens junctions, but may relax tight junctions and consequently the overall strength of homotypic cell-cell interactions. In fact, the presence of E-cadherin expression on cell-cell contacts indicated that adherens junctions in Rb knockdown cells still maintain substantial cell cohesion (Figure S2B). In vivo, Rb knockdown slightly stimulated expression of basal markers p63 and cytokeratins 5/6 and inhibited expression of E-cadherin and luminal cytokeratins 8/18 (Figure 2G). The de-repression of basal cytokeratins did not occur in vitro (Figure 2F and S2A), suggesting that the combination of Rb knockdown and in vivo factors synergized in shifting the differentiation profile of MCF7ras cells towards a more basal state. Same and/or different factors are potentially involved in upregulation of another basal marker, CD44, whose expression is increased three-fold in vivo (Figure 2G and S2C) but not in vitro (Figure 2F).

**Figure 3:**
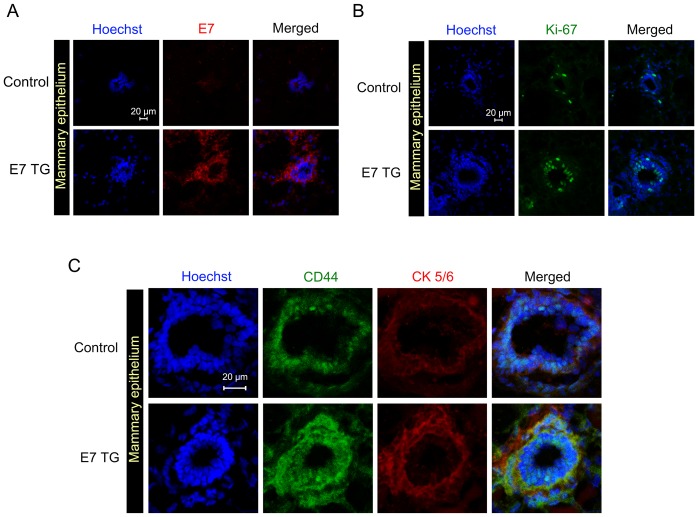
Suppression of Rb in basal mammary epithelium of E7 transgenic mice upregulates CD44 expression. Immunofluorescent images of sections from mammary epithelium of E7 transgenic and control mice. Staining with antibodies to (A) E7, (B) Ki-67, and (C) CD44 and cytokeratins 5/6 was performed as described in the Materials and Methods section. Scale bar, 20 μm.

To determine the role of Rb in regulating CD44 expression in vivo in normal basal mammary epithelial cells we used transgenic mice expressing human papilloma oncoprotein E7 that functionally inactivates proteins of Rb family (Rb, p107 and p130) [19]. E7 was expressed in basal mammary epithelium using a keratin 14 promoter. We noticed significant overexpression of E7, Ki-67, CD44 and cytokeratins 5/6 in the basal/myoepithelial layer of normal ducts (Figures 3A–3C) of E7 transgenic mice compared to control mice. These results indicate that Rb or its homologue (p107 or p130) acts as a repressor of CD44 expression and basal phenotype in normal mammary epithelium in vivo.

To address the role of Rb in BLCs we performed bioinformatic analyses of previously published gene expression data sets [20]. We confirmed that Rb mRNA expression is downregulated in 50% of BLCs [5], [20]. We also determined that CD44 is overexpressed in 75% of BLC specimens and that the increase in expression compared to all other breast cancer specimens is statistically significant (Figure S3A–B). In addition, we noticed that Rb levels were positively correlated with expression of genes, whose protein products are essential for epithelial integrity, e.g. E-cadherin, claudin-7, ZO-3, but also with markers of luminal phenotype, cytokeratins 8/18. Conversely, Rb levels were negatively correlated with basal cytokeratins 5/6 and with transcript levels of Rho-related proteins involved in cell motility. Expression of mesenchymal marker vimentin was not significantly correlated with Rb, but Slug (Snail-2) and Twist-1 were. Most of these correlations indicated that in human BLC tumors Rb potentially acts as a negative regulator of CD44 expression and basal phenotype, and a positive regulator of epithelial integrity, which is consistent with our experimental findings (Figure 2G).

Together, our results suggest that Rb suppression stimulates collective rather than single cell-based invasion and migration. Moreover, it induces several tumor characteristics typical of BLCs: extratumoral lymphovascular invasion, hematogenous metastatic spread, and increased expression of basal cytokeratins and CD44 in vivo.

### CD44 expression is essential for CCM stimulated by Rb knockdown in vitro

**Figure 4:**
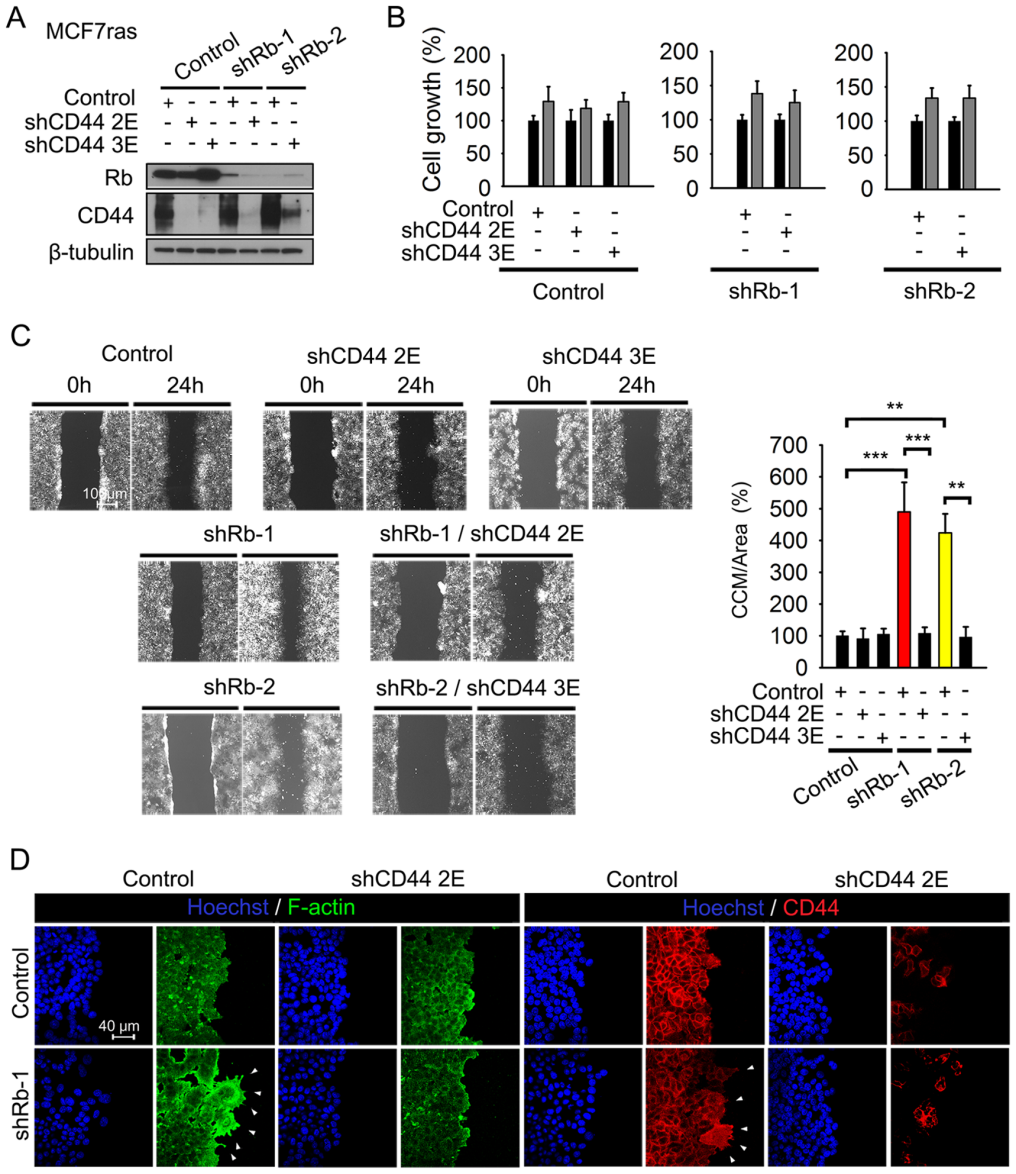
CD44 expression is essential for Rb knockdown-activated CCM in vitro. (A) MCF7ras cells were co-infected with a lentiviral construct expressing puromycin resistance gene and Rb or control shRNA. After selection, cells were infected with shRNA against CD44 or a scrambled sequence in an EGFP-expressing vector. Lysates from all double knockdown cells were examined by western blot with anti-CD44 and anti-Rb antibodies. β-tubulin was used as a loading control. (B) Growth of MCF7ras cells with double knockdown of Rb and CD44 was analyzed by MTT assay, *n* = 3 experiments, data are presented as mean ± SD. Controls included single Rb knockdowns, and cells with shRNA against scrambled sequence with or without further infection with CD44 shRNA. (C) CCM of double knockdown cells. The analysis was performed as in Figure 1B. Data are presented as mean ± SD. The experiment was performed three times in triplicate. Scale bar, 100 μm; unequal variance Student's t-test, ** p<0.01, *** p<0.001. (D) Immunofluorescent analysis of F-actin, CD44 and E-cadherin expression in cells with single and double Rb/CD44 knockdowns. Scale bar, 40 μm.

To determine if CD44 is an essential effector of CCM activated by Rb knockdown, we inhibited CD44 expression in MCF7ras cells expressing Rb shRNAs using two independent lentiviral constructs against CD44. We confirmed suppression of both Rb and CD44 in single and double knockdowns by western blot (Figure 4A) and tested cell growth and CCM (Figures 4B and 4C). Both shRNAs to CD44 eliminated CCM initiated by Rb inactivation without altering cell growth characteristics, indicating that CD44 is essential for CCM. To assess whether gain of CD44 function could by itself promote CCM in cells expressing endogenous levels of Rb, we attempted to overexpress two different isoforms of CD44- standard CD44s and epidermal/epithelial CD44v2–10 isoform. While the overexpression of CD44v2–10 was lethal (not shown), overexpression of CD44s was tolerated, but resulted in SCM and not in CCM (Figures S4A and S4B). It also induced partial EMT phenotype characterized by downregulation of E-cadherin protein expression and activation of Akt (Figure S5A). This phenotype is consonant with already described important role of CD44s in EMT-dependent single cell-based migration [21].

Interestingly, Rb knockdown also led to pronounced extension of F-actin positive and CD44 positive filopodia-like structures that were significantly less pronounced in control cells (Figure 4D and S6). Several studies have recently shown that extended cellular protrusions, such as filopodia and invadopodia, are essential for initiation and progression of metastasis [22], [23]. Importantly, the protrusions were eliminated by CD44 knockdown. Finally, E-cadherin expression at adherens junctions (Figure S2B) suggested that Rb knockdown cells maintain their epithelial-epithelial cell contact and migrate in collective manner. These experiments provided a clear rationale for repression of CD44 expression by Rb that we observed in vivo: Rb suppresses CD44 activity to inhibit CCM.

### CD44 expression is essential for lymphovascular and mammary fat pad invasion, and for lung metastasIs initiated by Rb knockdown

**Figure 5:**
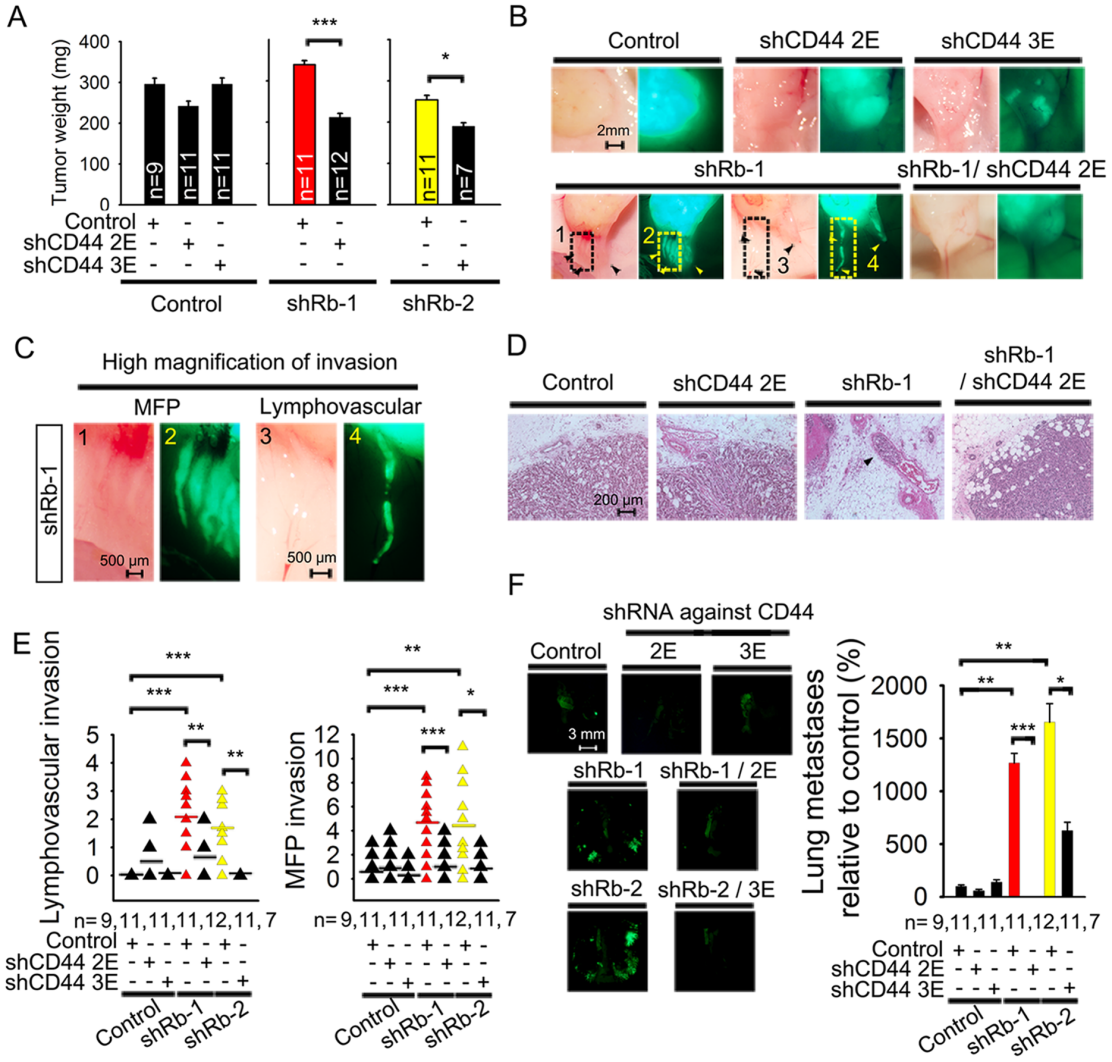
CD44 expression is essential for lymphovascular invasion and lung metastases initiated by Rb knockdown. (A) Weight of orthotopic primary tumors initiated by MCF7ras cells with double knockdown of Rb and CD44 eight weeks after mammary fat pad injection. Rb shRNAs and one of the control shRNAs were expressed in a vector conferring puromycin resistance. CD44 shRNAs and the second control shRNA were expressed in an EGFP vector. Data are presented as mean ± SD, *n* is the number of mice in each group. Equal variance Student's t-test, * p<0.05, *** p<0.001. (B) Representative phase contrast and fluorescent images of tumor-attached EGFP-positive cancer cells/clusters invading mammary fat pad or adjacent capillaries. Analysis was performed on the whole animal post mortem. Scale bar, 2 mm. (C) Representative high magnification phase contrast and fluorescent images of Rb-1 shRNA from Figure 5B with visible MFP and lymphovascular invasion. Scale bar, 500 μm. (D) H&E staining of primary tumors from mice injected with Rb and CD44 double knockdown cells. Arrows indicate areas of lymphovascular invasion. Scale bar, 200 μm. (E) Quantification of collective cell invasion into host mammary fat pad and extratumoral capillaries. Mammary fat pad invasion was quantified as incidence of EGFP-positive cell clusters in the area adjacent to tumor and not within a capillary (as judged by presence of erythrocytes in the phase contrast image). Cell clusters considered to be within a capillary were counted as lymphovascular invasion. Number of animals in each group is indicated as *n*. Unequal variance Student's t-test, * p<0.05, ** p<0.01, *** p<0.001. (F) Fluorescent images and quantification of lung metastases' numbers initiated by primary tumors 8 weeks after implantation of MCF7ras cells with double knockdown of Rb and CD44 into mammary fat pad. Number of metastases were quantified by ImageJ and normalized to the weight of primary tumor; number of animals in each group is indicated by *n*. Scale bar on fluorescent images, 3 mm. Unequal variance Student's t-test, * p<0.05, ** p<0.01, *** p<0.001.

To determine the role of CD44 in lymphovascular and MFP invasion present in Rb knockdown tumors, we implanted MCF7ras cells with stable single and double Rb/CD44 knockdowns into mouse MFPs and analyzed the effect of CD44 inactivation on primary tumor growth and metastatic progression. The single Rb and CD44 knockdowns did not significantly alter primary tumor weight (Figure 5A). However, double knockdown tumors were about 30% (shRb-1/shCD44–2E) or 20% (shRb-2/shCD44–3E) lighter than Rb knockdown alone, suggesting that CD44 suppression has a strong impact on tumor growth in the context of Rb suppression.

Next, using low magnification fluorescent microscopy, we determined the role Rb and CD44 play in the invasion of primary tumors into capillaries and MFPs. We noticed that both Rb knockdowns displayed significant macroscopic lymphovascular (1.9 or 1.2 invading strand per mouse, respectively) and MFP (4.6 or 4.3 invading strands per mouse) invasion (Figures 5B and 5C, Figure 5E and Figures S7A–S7C), which extended up to 5 mm in length. This was in agreement with the lymphovascular and MFP invasion detected by histopathological analysis (Figure 2B). Moreover, some cell clusters seemed to detach from the rest of the invasion strand, indicating a possible cellular mechanism for release of cell clusters into the circulation. Both double knockdown Rb/CD44 cell lines had suppressed lymphovascular (5.5 fold or more) and MFP (3.6 or 3.7 fold) invasion (Figures 5D–5E and S7D–S7F) compared to single Rb knockdowns, suggesting that CD44 is essential for both types of collective invasion in vivo. However, it is possible that lymphovascular invasion was underestimated for two reasons: A) We took pictures of only one plane and B) some or all of the strands identified as MFP invasion might in fact have originated as lymphovascular invasion.

Despite relatively low numbers of detected invading strands, Rb knockdowns initiated massive (1263% or 1653% metastases per lungs vs. 100% in controls) metastatic growth in lungs as evidenced by dissecting fluorescence microscopy pictures (Figure 5F) and validated by histopathological analysis of hematoxylin and eosin (H&E) stained specimens (Figures S7G and S7H). This phenotype was CD44-dependent and consistent with the collective invasion observed in the initial shRb knockdown experiment. Altogether these findings strongly implicate that CD44 is an essential effector for Rb knockdown-induced lymphovascular and MFP invasion as well as for lung metastases.

### Rb/CD44-dependent release of circulating single cancer cells (CCC^S^) and circulating clustered cancer cells (CCC^C^) from primary tumor

The release of CCC with intact cell membranes into the hematogenous circulation (Figure 2E) suggested that Rb suppresses metastatic progression also at the level of dissemination. We wished to determine the expression and role of CD44 in this process as well as in the size distribution of CCC. We therefore isolated CCC from mice bearing primary tumors with single and double knockdowns of Rb and CD44.

**Figure 6:**
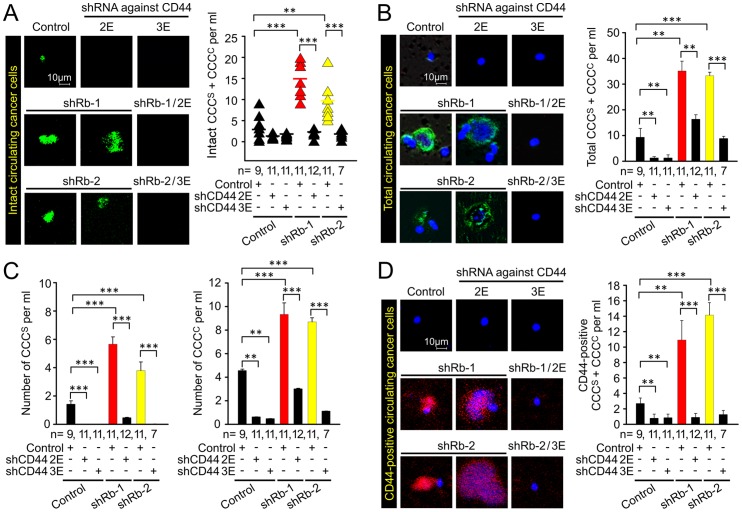
Regulation of incidence of circulating single/clustered cancer cells (CCC^S^/CCC^C^) by Rb and CD44. (A) Fluorescent images and quantification of circulating live cancer cells and cell clusters co-isolated with mononuclear fraction from blood of animals bearing tumors. The tumors were induced by orthotopic implantation of MCF7ras cells expressing shRNAs against Rb, CD44, and their combination, along with respective controls. EGFP is expressed in cytoplasm and its presence indicates both cancer cell identity and presumably intact membrane. Number of animals in each group is depicted on the graph. Scale bar, 10 μm; unequal variance Student's t-test, ** p<0.01, *** p<0.001. (B) Fluorescent images and quantification of total circulating cancer cells (both alive and dead) detected with a human-specific antibody to a nuclear antigen. Data are presented as mean ± SEM. Scale bar, 10 μm; unequal variance Student's t-test, ** p<0.01, *** p<0.001. (C) Number of CCC^S^ and CCC^C^ in the total number of cancer cells detected with a human-specific antibody to a nuclear antigen. Data are presented as mean ± SEM; unequal variance Student's t-test, ** p<0.01, *** p<0.001. (D) Fluorescent images and quantification of CD44 positive cancer cells detected with a human-specific anti-CD44 antibody. Data are presented as mean ± SEM. Scale bar, 10 μm; unequal variance Student's t-test, ** p<0.01, *** p<0.001.

The single Rb knockdown tumors released 14.7 (shRb-1) or 9.6 (shRb-2) cells and cell clusters (CCC^S^ + CCC^C^) with intact membranes per ml of blood, while control tumors released 3.5 cells and cell clusters per ml (Figure 6A). Inactivation of CD44 expression suppressed the numbers of CCC by 78–80% for controls and 84–87% in Rb knockdowns. We concluded that loss of Rb function stimulated the release of presumably viable cancer cells with intact membranes (EGFP-positive) into the circulation in a CD44-dependent manner.

Loss of endogenous CD44 in Rb expressing cells results in decreased phosphorylation of Rb at S780, but not S608 and S807/811 (Figure S5). On the other hand, gain of CD44s resulted in slight downregulation of S608, upregulation S807/811 and change in S780 (Figure S5). These results indicate potential relationship of Rb phosphorylation with CD44 expression in cells with intact Rb levels.

Several previous reports suggested that CCC in cancer patients are detected not only as single cells but also as cell clusters and cell fragments without nuclei (cell ghosts) [24], [25]. Therefore, we determined the frequencies of CCC^S^, CCC^C^ (containing two or more cancer cells) and cell ghosts with a human-specific antibody against a nuclear antigen. We found that Rb knockdown tumors released about four times more cancer cells, clusters and ghosts combined than controls (Figure 6B). They also released 2–3 times more CCC^S^ and about 2 times more CCC^C^ than tumors expressing control shRNA (Figure 6C). Release of total CCC, CCC^S^ and CCC^C^ was strongly suppressed in double Rb/CD44 knockdown cells (Figures 6A–6C).

Next, we enumerated the frequency of human CD44-positive circulating cells and clusters. It has been shown that about one third of CCC in breast cancer patients are of CD44^high^/CD24^low^ phenotype [26]. We detected about 10.9 (shRb-1) or 14.1 (shRb-2) CD44-positive circulating cancer cells/clusters per ml of blood, suggesting that the majority of CCC released from Rb knockdown tumors express CD44 while the control released only 2.5 CD44-positive CCC per ml of blood (Figure 6D). This is consistent with higher expression of CD44 protein in shRb-1 and shRb-2 primary tumors (Figure S8A). The loss of Rb increased the total number of CD44-positive CCC and total CCC; however, the relative numbers of CD44-positive CCC compared to the overall intensity of CD44 expression in primary tumors remain similar (Fig. S8B). These results strongly suggest that de-repressed CD44 in Rb knockdown cells in vivo is essential not only for initial lymphovascular invasion but also for the release of viable cancer cells into the circulation.

**Figure 7:**
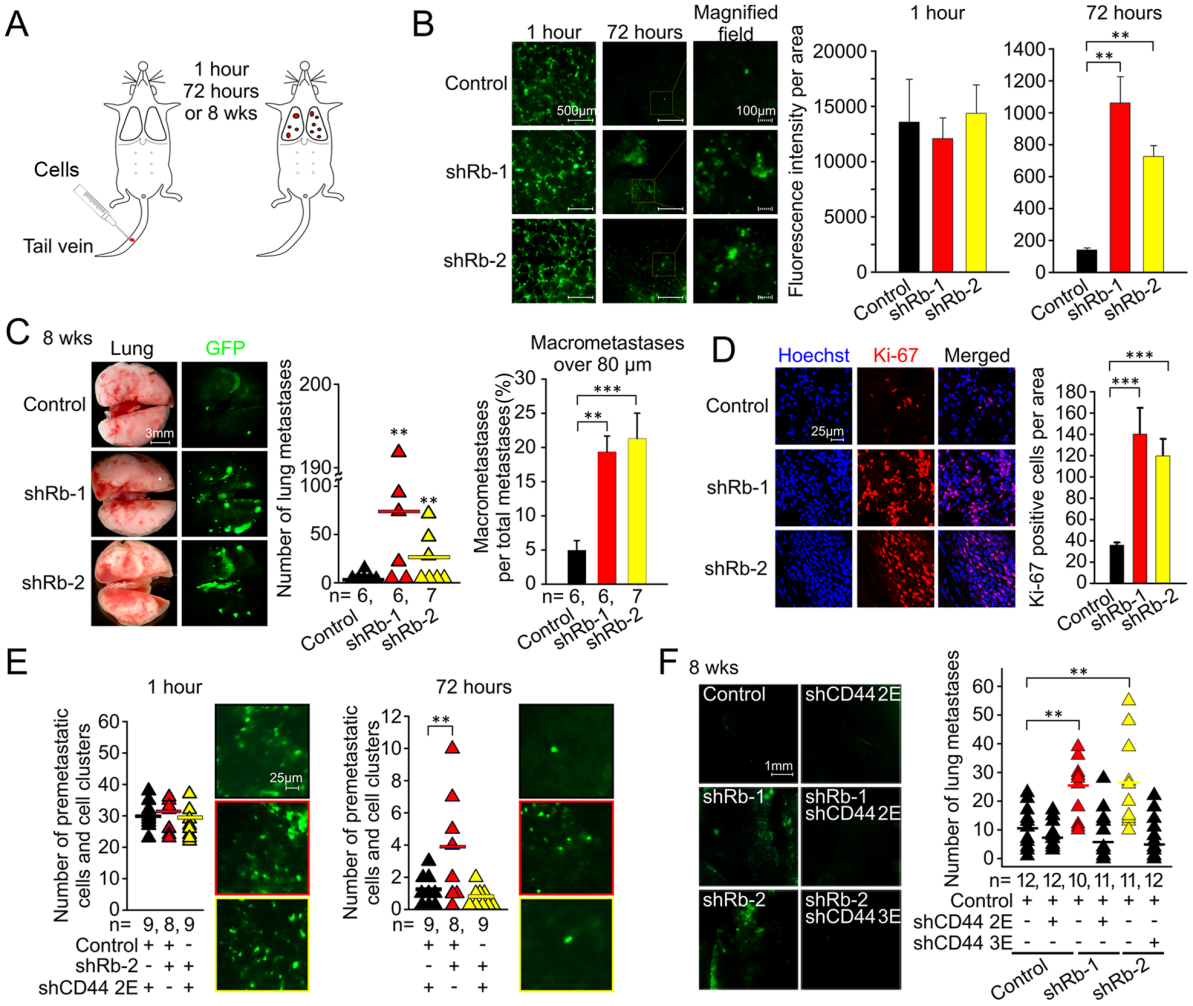
Rb/CD44-dependent regulation of initial cell survival and metastatic progression. (A) Illustration of tail vein injection experiment in mouse. (B) MCF7ras cells were infected with shRNA to Rb or control sequence expressed in an EGFP vector. The resulting cells were injected in tail vein and fluorescent images of whole lungs were taken at indicated time points. Representative images are shown. Total fluorescence of whole lung images was quantified and is plotted as mean ± SD. Scale bar, 500 μm (left and middle panels) and 100 μm (right panel); equal variance Student's t-test, ** p<0.01. (C) Metastatic-like tumor growth in lungs 8 weeks after tail vein injection. Total number of metastases quantified by ImageJ is plotted into the graph on the left. The ratio of macrometastases to the total number of metastases in each pair of lungs is presented in the graph on the right. The number of animals in each group is indicated as *n*. Data is shown as mean ± SEM. Scale bar, 3 mm; equal variance Student's t-test, ** p<0.01, *** p<0.001. (D) Immunofluorescence of frozen sections from lungs of mice with metastatic growth after tail vein injection. Number of Ki-67 positive cells was quantified by ImageJ and is presented as mean ± SD. Scale bar, 25 μm; equal variance Student's t-test, *** p<0.001. (E) Dissecting microscope fluorescent images and quantification of cells/cell clusters in lungs one hour or 72 hours after tail vein injection of MCF7ras cells with suppressed Rb and/or CD44 expression. Representative pictures are shown. Scale bar, 25 μm; unequal variance Student's t-test, ** p<0.01. (F) Whole lungs fluorescent images from metastatic tumor growth eight weeks after tail vein injection of single/double Rb/CD44 knockdowns. Number of metastatic foci per mouse was quantified by ImageJ and is plotted in the graph. Scale bar for fluorescent images, 1 mm; unequal variance Student's t-test, ** p<0.01.

To finalize the analysis of multistage metastatic progression of MCF7ras cells with suppressed Rb expression, we determined the role of the Rb/CD44 pathway in early survival, metastasis-initiation potency, and active growth of metastases in lungs. This was accomplished by implanting MCF7ras cells into lungs via tail-vein injection and subsequent quantitation of lung metastases (Figure 7A). The inactivation of Rb expression did not alter initial survival in lungs one hour after tail-vein injection, but it increased the survival 72 hours post-injection by a factor of four (Figure 7B). This early survival correlated with 2.5-fold higher metastasis-initiating ability of cells with Rb knockdown (Figure 7C). In addition, Rb knockdown stimulated highly proliferative later stages of metastatic growth as evidenced by the increase of macrometastatic fraction of metastases and strong Ki-67 positivity of metastatic lesions (Figures 7C and 7D). The suppression of CD44 in Rb knockdown cells eliminated their ability to initially survive and/or be retained in lungs as well as their potential to form metastases after eight weeks (Figures 7E and 7F). Hence, the Rb/CD44 pathway is essential regulator of early as well as of the late stages of metastatic progression.

## Discussion

Here we show that Rb inactivation is sufficient to activate multiple steps in the metastatic cascade including CCM in vitro, lymphovascular and MFP invasion, release of cancer cell clusters and single cells into the circulation, and the growth of metastatic tumors in lungs. Moreover, we demonstrate that inactivation of Rb stimulates CD44 expression in vivo and that CD44 expression is essential for all the above-mentioned phenotypes. We speculate that in vitro Rb inactivation may activate CCM through stimulating expression of known CD44 effector molecules such as Rho A and C. Ongoing studies focus on specifically addressing the downstream events that trigger CCM.

Collective cell movement is typical for mammary development when terminal end buds extend to form the whole mammary ductal structure [3], and a part of this migration machinery is possibly reactivated by suppression of Rb expression in cancer cells. A high-throughput screen for regulators of breast epithelial cell migration in the scratch-assay format discovered that 14 out of 66 active genes stimulated or inhibited CCM, suggesting that CCM is an important mode of epithelial cell migration in vitro [27]. Previous work has also established that mammary epithelial cells can gain survival and migratory abilities by losing the epithelial phenotype and simultaneously acquiring mesenchymal phenotype via EMT [28], [29], and that partial EMT can potentially facilitate collective cell migration [30].

We observed that Rb knockdown may result in partial EMT in MCF7ras cells as indicated by increased expression of the EMT-inducing transcription factor, Snail, and by downregulation of essential tight junction proteins ZO-1 and claudin-1. Previously published literature suggested that Rb suppression results in inhibition of adherens junction protein E-cadherin in MCF-7 cells, which visually appeared as partial EMT [31]. In addition, overexpression of Rb led to restoration of epithelial phenotype [31], [32] in mesenchymal cells. In our model, E-cadherin was downregulated in vivo and in vitro in ZR-75 but not in other cell lines, suggesting that E-cadherin regulation by Rb is dependent on cellular context. However, based on the expression of the other EMT markers, partial EMT can possibly facilitate migratory ability of Rb knockdown cells while still keeping them attached to each other. The expression of vimentin does not seem to determine the mode of migration as it is downregulated in Rb knockdown of MCF7ras cells and overexpressed in Rb knockdown of MCF7 and MCF10A cells without altering the proclivity of these cells to migrate collectively rather than singly.

In the in vivo setting we cannot exclude that lymphovascular invasion originates from a single invading cancer cell that subsequently creates an invading cell cluster. However, the detached cluster geometry and strong presence of CCM in the MFP support our hypothesis that lymphovascular invasion begins as CCM.

Recent evidence suggests that the process of intravasation results in a plethora of CCC combinations: alive and dead single cancer cells, cancer cell clusters, and both combined with host stromal cells [33], [34]. This indicates that the process of release of cancer cells into circulation can be caused by a succession of two events. The initial event is lymphovascular invasion induced in our case by Rb knockdown. The following one is likely a fragmentation of invading strands by a complex mechanism consisting of EMT, necrosis/apoptosis, or combination of these events, that ultimately leads to the release of a complex mixture of viable and non-viable single cells and cell clusters. Such mechanism is consistent with high frequency of dead CCC we observed in the circulation and the fact that CCC from breast cancer patients are often apoptotic [35].

Suppression of Rb activated several characteristics typical of BLCs, namely expression of basal markers (cytokeratins 5/6, p63 and CD44), lymphovascular invasion, and lung metastases. Lymphovascular invasion seems to be a consequence of increased collective cell motility detected in vitro. This is reinforced by the notion that both phenomena are CD44-dependent in vitro and in vivo.

Rb is known to inhibit cell differentiation [36], which is consistent with the de-differentiation-like phenotype in Rb knockdown cells, namely the decrease of luminal markers cytokeratins 8/18 and increase of basal markers p63 and cytokeratins 5/6 in vivo. While cytokeratins 8/18 in our experiments are downregulated in Rb knockdown cells also in vitro, the increase in basal phenotype (cytokeratins 5/6, p63 and CD44) is noticeable in vivo, suggesting that inactivation of Rb continues to require co-stimulatory signal(s) from host stroma to induce partial de-differentiation of MCF7ras cells into a more basal phenotype. Such collaboration between genetic alterations facilitated by in vivo factors is similar to a previous report describing CCM of TGFβ-RII^−/−^ breast cancer cells activated in the presence of stromal cells [37].

The basal/CD44-high in vivo phenotype of Rb knockdowns was consistent with metastasis-initiating potency of MCF7ras cells with Rb knockdown and its CD44-dependent nature. Both phenomena also suggested that CD44 is not only a marker but an essential component of metastasis-initiating cells (metastatic cancer stem cells) in BLCs. This is an extension of our previous findings that CD44 is essential for primary tumor-initiating ability in cells with Rb or p53 tumor suppressor inactivation by SV40 large T antigen [13].

Because BLCs are a particularly aggressive and drug-resistant subset of breast carcinomas [38], targeting the Rb-low subsets with anti-CD44 therapy can potentially overcome the drug resistance. In fact, anti-CD44 therapy recently became a possibility, when humanized CD44 antibodies were developed by multiple pharmaceutical companies and tested in both preclinical and clinical trials [39], [40]. These antibodies were effective against primary tumor growth, but their effect on metastases is not known. Based on the work presented here, we think that treatment targeting CD44 with antibody or other approaches has the potential to become important therapy for invasive and metastatic basal-like carcinomas, and perhaps other subsets of breast carcinomas.

**Figure 8:**
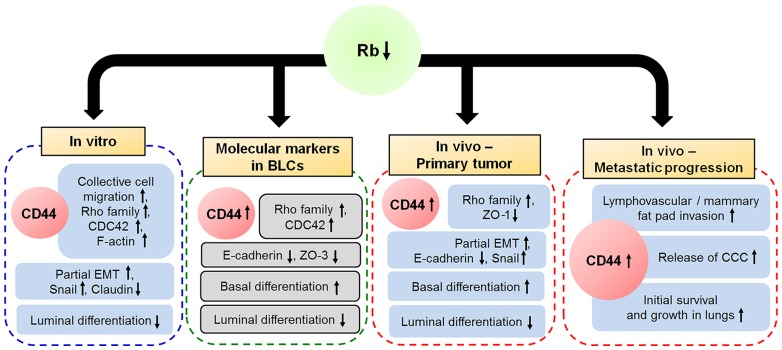
Model of Rb-CD44 axis in metastatic progression. Rb/CD44 regulate CCM, lymphovascular and mammary fat pad invasion, release of CCC and initiation and progression of metastasis in breast tumorigenesis. Inactivation of Rb suppresses epithelial integrity and promotes CD44-dependent CCM in vitro. In vivo, both loss of Rb and presence of CD44 are required for mammary fat pad and lymphovascular invasion, release of CCC into the blood stream, early survival, and subsequent metastatic growth in lungs. Clinical expression data suggest that Rb expression is suppressed and CD44 is upregulated in BLCs. In addition low Rb expression correlates with higher expression of basal markers, low expression of luminal markers, low expression of markers of epithelial integrity and high levels of Rho family proteins involved in cell motility and with.

In conclusion, our current results suggest that Rb suppresses lymphovascular invasion, release of CCC, and metastasis by inhibiting CCM. This mechanism is dependent on expression of CD44, which is essential not only for the metastasis initiation ability of Rb knockdown cells, but also for lymphovascular and MFP invasion, release of cancer cells into the circulation and growth of metastases (Figure 8). This phenotype is a testimony of the critical and complex role of Rb/CD44 pathway in suppression of metastatic progression and a promising target for anti-BLC therapy.

## Materials and Methods

### Cell Culture and Viral Transduction

Human breast carcinoma cell lines MCF7, MCF7ras, ZR-75, and T47D were maintained in RPMI or DMEM supplemented with 10% heat inactivated fetal calf serum, 100 units/ml penicillin, 100 μg/ml streptomycin, and 2 mM L-glutamine at 37°C in 5% CO_2_. MCF10A cells were cultured in DMEM supplemented with 5% horse serum, 10 µg/mL insulin, 20 ng/mL recombinant human EGF, 0.5 µg/mL hydrocortisone, 100 units/ml penicillin, 100 μg/ml streptomycin, and 2 mM L-glutamine at 37°C in 5% CO_2_. The lentiviral constructs of shRNAs to Rb (shRNAs Rb-1 and -2) in pLKO1-Puro were from Addgene (plasmids 25640 and 25641) and the lentiviral constructs of shRNAs to CD44 (shRNA CD44-2 and -3 and control scrambled shRNA (shLuc)) were used as described previously. [13] The three- plasmid system was used to produce lentiviral particles.

### Antibodies

For immunohistochemistry, we used anti-CD31 antibody (Abcam, ab28364). For immunofluorescence analyses, we used phycoerythrin (PE)-conjugated anti-CD44 antibody (Becton Dickinson, Nr.550989), anti-F-actin antibody (Becton Dickinson, 612657), a human-specific antibody to a nuclear antigen (Antibodies, ABIN361360), anti-E7 (Santa Cruz, sc-6981), and anti-Ki-67 (Santa Cruz, sc-7846) antibodies.

For immunofluorescence and/or western blot analyses of p53 and E-cadherin, we used rabbit anti-p53 antibody (Santa Cruz, FL-393) and mouse monoclonal antibody to E-cadherin (Santa Cruz, sc-21791). Alexa fluorescent secondary antibody conjugates were from Invitrogen.

For western blot analysis of CD44 expression, we used anti-CD44H antibody (R&D Systems). Antibodies for western blot detection of Rb, E2F1, ZEB1, Snail, Rho A, Rho B, Rho C, CDC42, ZO-1, Akt and p-ERK were purchased from Cell Signaling (Danvers, MA, USA); cytokeratins 5/6 antibody was purchased from Millipore (Temecula, CA, USA) and cytokeratins 8/18 antibody was purchased from DakoCytomation (Glostrup, Denmark).

### Gene expression profiling analysis and identification of molecular subtypes

The data used for expression profiling were from ArrayExpress (http:www.ebi.ac.uk/arrayexpress/). Breast cancers were classified by assigning each tumor to the subtype defined by hierarchical clustering of expression pattern as described previously (Perou et al., 1999). Hierarchical clustering analyses were performed using Cluster 3.0 (http://bonsai.hgc.jp/~mdehoon/software/cluster/software.htm) on median centered log expression values using Pearson correlation and centroid linkage. Clustered data were visualized using Java TreeView (http://jtreeview.sourceforge.net/). Pearson coefficient was used to analyze correlation of Rb expression with other genes in all 130 specimens.

### Mammosphere assay

Mammosphere culture was performed as previously described. [41] Cells were plated at 20,000 cells/ml and grown in 1% methylcellulose on poly–HEMA coated 6-well plates in DMEM supplemented with 10% serum and 20 ng/ml hEGF. Suspension cultures were cultured for 14 days. Then, microscope images were taken by phase-contrast microscope and mammosphere-forming ability was quantified by ImageJ (NIH).

### Cell Growth Assay

MCF7, MCF7ras, ZR-75, and MCF10A cells (2×10^4^) were plated on 96-well plates. Cells were allowed to grow for 24 hours and the resulting growth was detected using an MTT assay (Alfa Aesar, L11939).

### Collective Cell Migration Assays

Cells (1×10^5^) were plated on 22×22 mm cover slips in 6 well plates and allowed to proliferate for 48 hours. Confluent monolayers were gently scratched using a 200 μL pipet tip. Cells at partially detached edges of the scratch were allowed to reattach for additional 3 hours. Then, microscope images were taken and defined as initial point of CCM (T = 0 hours). Cells were allowed to migrate for 24 hours and the resulting CCM was analyzed by fluorescent microscopy and quantified by ImageJ (NIH) or Photoshop CS5 (Adobe Systems).

### Single-Cell Migration Assay

Cells (1×10^5^) were plated on 8 μm pore size Transwell filters in either DMEM or RPMI medium and allowed to migrate for 24 hours. The transmigrated cells were detected with Hoechst fluorescent dye and counted.

### Immunofluorescence

The cells or tissues were washed with phosphate-buffered saline (PBS), permeabilized and fixed in 100% methanol at 4°C for 30 minutes. The cells or tissues were washed three times and incubated with the blocking solution (5% BSA in PBS). The cells were then incubated with the primary antibodies for 2 hr or overnight, washed three times with PBS containing 0.1% Tween-20 for 15 minutes, and finally incubated with secondary antibodies (Invitrogen) and Hoechst stain to detect nuclear DNA (Invitrogen, H1399) for 30 minutes. The slides were washed extensively with PBS and mounted with Immu-mount (Fisher Scientific). All matched samples were photographed (control and test) using immunofluorescence microscope (Zeiss, LSM710) and stained sequentially with primary rabbit antibodies, followed by incubation with secondary antibodies conjugated to Alexa-488 or Alexa-598. They were then incubated with Hoechst stain to detect nuclear DNA (Invitrogen, H1399).

All pictures were taken with the same exposure conditions without autoscaling.

### Animals and Surgery

All mouse experiments were conducted in facilities approved by the Laboratory Animal Medical Services and all procedures were approved by Institutional Animal Care and Use Committee at the University of Cincinnati. NOD/SCID mice were bred in house. Mice were 6–8 weeks of age at time of injections. The MCF7ras cells (1×10^6^) were injected into mammary fat pad in 50 µl growth medium. Each group consisted of 12 mice. The mammary tumors were dissected at the end of the experiment and weighed. EGFP-positive lung metastases visualized by fluorescent microscopy were counted manually or with ImageJ software. Tissue samples were fixed in 10% buffered formalin for 12 h, followed by a wash with PBS and transfer to 70% ethanol, and then embedded in paraffin, sectioned and stained with haematoxylin and eosin.

For direct deposition into lungs, MCF7ras cells (1×10^6^) were injected into tail vein.

### Statistical Analysis

All of the data are presented as mean ± standard deviation (SD) or mean ± standard error of the mean (SEM). Statistics was performed using an equal variance or unequal variance Student's t-test. Statistical significance was accepted at a level of p<0.05. All analyses were performed using SAS software (version 9.3; SAS Institue, Cary, NC, USA).

## Acknowledgments

We thank S.C. Wang for MCF7 cells, N. Ben-Jonathan for MCF10A and T47D cells and S. Waltz for allowing us to use her dissecting microscope. We thank M. Czyzyk-Krzeska and S. Khan for reading the manuscript and providing valuable comments; B. Ehmer and G. Doerman for excellent technical assistance; and B.E. Peace for editorial assistance.

## Supporting Information

Figure S1 **Inactivation of Rb promotes CCM but not SCM in MCF7ras cells.** (A) Quantification of CCM of MCF7ras breast cancer cell line. Cells were infected with lentivirus encoding shRNA against Rb or control sequence. CCM was quantified as an area covered during 24-hour migration, and expressed as a percentage relative to the control. The experiment was performed three times in triplicate. Data are presented as mean ± SD. Scale bar, 100 μm; equal variance Student's t-test, ** p<0.01. (B) Quantification of SCM assays. MCF7ras cells expressing shRNA against Rb or control sequence were allowed to migrate for 24 hours. The experiment was performed three times in triplicate. Data are presented as mean ± SD. (C) MCF7ras breast cancer cell line derivatives were analyzed 24 hours after seeding by MTT assay. Data from a representative experiment (n = 5) performed in triplicate are expressed as amount of metabolized MTT measured by absorbance normalized to the absorbance of control shRNA and presented as mean ± SD. (D) Phase contrast images of mammosphere-forming potency of MCF7ras and T47D cells with Rb knockdown. Arrows indicate protrusions formed by invading cells and cell clusters. Scale bar, 50 µm.
(TIF)

Figure S2 **Breast cancer cell lines with Rb knockdown undergo partial EMT.** (A) Western blot of cell lysates from MCF7, MCF10A, and ZR-75 cell lines expressing control or Rb shRNA. β-tubulin was used as a loading control. (B) Immunofluorescence image of Rb knockdown MCF7ras cells stained with antibody against E-cadherin (green) and with Hoechst (blue). Scale bar, 20 μm. (C) Quantification of CD44 expression in vivo presented in Figure 2G. Data are depicted as mean ± SD; equal variance Student's t-test, * p<0.05.
(TIF)

Figure S3 **Analysis of mRNA expression data from different types of primary human breast cancers.** (A) Comparison of Rb and CD44 mRNA expression in basal-like, ERBB2, luminal A, luminal B, and normal like type of breast cancer. Percentage indicates the fraction of given tumor type featuring Rb-low or CD44-high level. (B) Expression of CD44 mRNA in BLCs versus CD44 mRNA in all other breast cancer specimens. Unequal variance Student's t-test, * p<0.05. (C) Pearson's correlation of Rb expression with CCM related genes, markers of epithelial to mesenchymal transition (EMT), tight junctions, and differentiation across all 130 breast cancer specimens.
(TIF)

Figure S4 **Overexpression of CD44s in Rb positive cells stimulates SCM but not CCM.** (A) Quantification of CCM of MCF7ras breast cancer cell line ectopically expressing standard isoform of CD44 or control cDNA. CCM was quantified as an area covered during 24-hour migration, and expressed as a percentage relative to the control. The experiment was performed three times in triplicate. Data are presented as mean ± SD. Scale bar, 100 μm. (B) Quantification of SCM assays. Cells overexpressing CD44 or control cDNA were allowed to migrate for 24 hours. The experiment was performed three times in triplicate. Data are presented as mean ± SD. Scale bar, 100 μm; equal variance Student's t-test, *** p<0.001.
(TIF)

Figure S5 **CD44 expression plays a role in Rb phosphorylation.** (A) Western blot of cell lysates from MCF7ras cell line ectopically expressing standard isoform of CD44 or control cDNA. β-actin was used as a loading control. (B) Western blot of cell lysates from MCF7ras cell line expressing control or CD44 shRNA. β-actin was used as a loading control.
(TIF)

Figure S6 **Loss of Rb leads to pronounced extention of F-actin positive filopodia-like formation during CCM.** Immunofluorescent analysis of F-actin expression in cells with single Rb knockdowns. Scale bar, 40 μm.
(TIF)

Figure S7 **Collective invasion and lung metastases induced by loss of Rb require CD44.** (A) Representative phase contrast and fluorescent images of EGFP-positive cancer cells/clusters invading mammary fat pad or adjacent capillaries from orthotopic primary tumor initiated by cells infected with shRNA to Rb and CD44. Analysis was performed on the whole animal post mortem. Scale bar, 2 mm. (B) High magnification phase contrast and fluorescent images of (A). Scale bar, 500 μm. (C) Staining of a sample from primary tumor with anti-CD31 antibody displaying lymphovascular invasion from primary tumor initiated by Rb knockdown cells. Scale bar, 20 μm. (D) Quantification of lymphovascular invasion from primary tumors based on fluorescent images of whole mice. The first number column in the table delineates sum of detected lymphovascular invasion events in the group followed by number of animals in the group. The incidence represents percentage of animals in the group with any detected lymphovascular invasion. (E) Quantification of mammary fat pad invasion from primary tumor based on fluorescent images of whole mice. For this quantification only cell clusters that were considered to be outside of capillary were counted. Sum of all MFP invasion events is followed by number of mice in each group, and percentage of animals in the group with noted event. (F) H&E staining of primary tumors from mice injected with Rb and CD44 double knockdown cells. Arrows indicate areas of lymphovascular invasion as judged by the presence of erythrocytes on the complementary phase contrast images. Scale bar, 200 μm. (G) Quantification of lung metastatic spread from fluorescent images of lungs. Data are expressed as total number of metastases for each group (quantified by ImageJ software) followed by the number of animals in each group. Incidence is the percentage of animals in the group with any detected metastases. (H) H&E staining of lung metastases. Scale bar, 200 μm.
(TIF)

Figure S8 **Loss of Rb induces CD44 expression and release of CCC** ***in vivo*** **.** (A) Immunofluorescence images and quantification of CD44 expression in Rb knockdown MCF7ras cells stained with antibody against CD44 (red) or Hoechst (blue). Scale bar, 0.2 mm; equal variance Student's t-test, ** p<0.01.(B) Quantification of total CCC and CD44-positive CCC relative to control, related to Figure 6B and 6D.
(TIF)
