# Supplementary figures and images for: Rb Suppresses Collective Invasion, Circulation and Metastasis of Breast Cancer Cells in CD44-Dependent Manner
Source: PLoS One. 2013 Dec 4;8(12):e80590. doi: 10.1371/journal.pone.0080590 (PMC3851742; doi:10.1371/journal.pone.0080590)

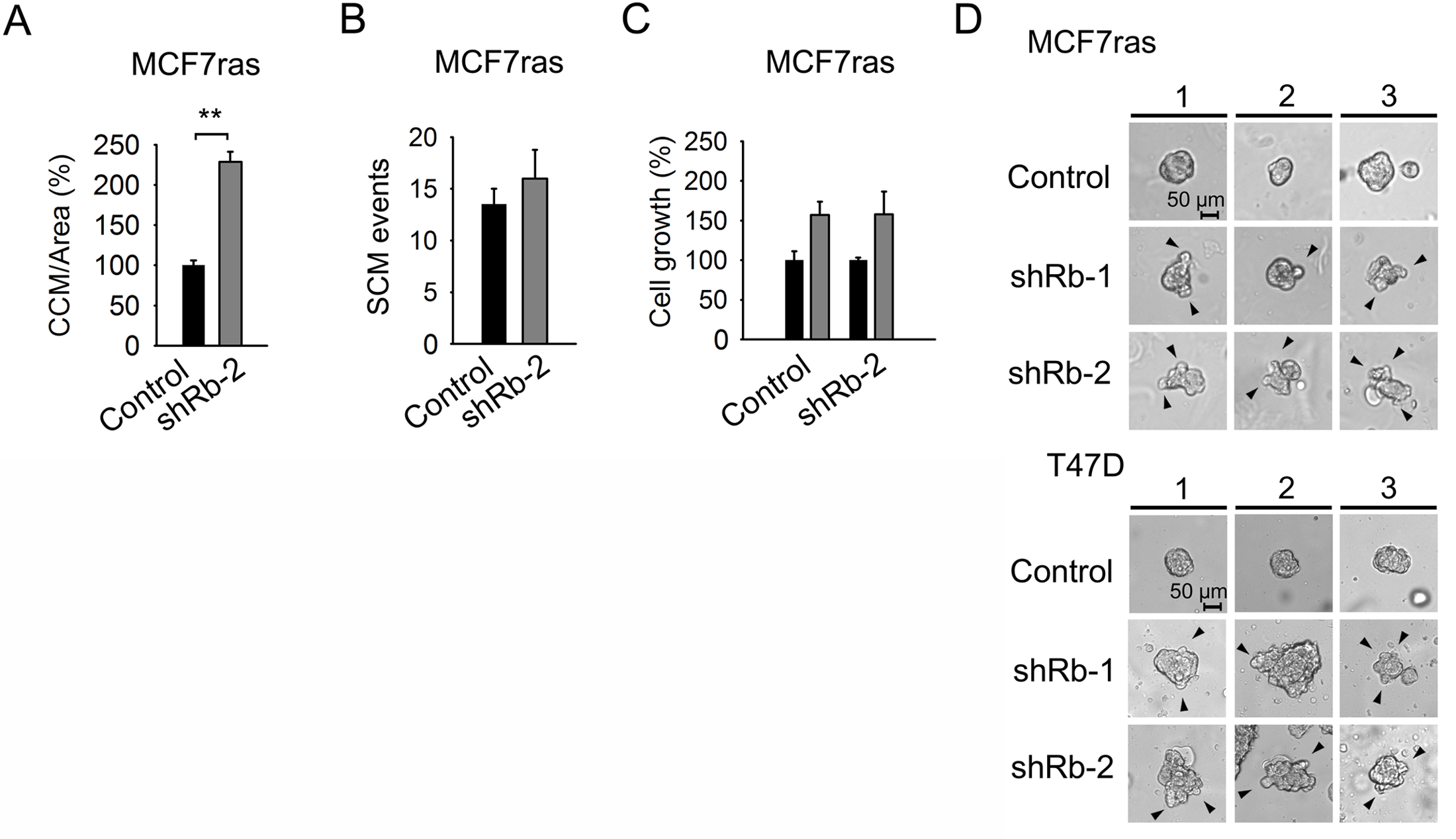

Supplement: Figure S1 — Inactivation of Rb promotes CCM but not SCM in MCF7ras cells. (A) Quantification of CCM of MCF7ras breast cancer cell line. Cells were infected with lentivirus encoding shRNA against Rb or control sequence. CCM was quantified as an area covered during 24-hour migration, and expressed as a percentage relative to the control. The experiment was performed three times in triplicate. Data are presented as mean ± SD. Scale bar, 100 μm; equal variance Student's t-test, ** p<0.01. (B) Quantification of SCM assays. MCF7ras cells expressing shRNA against Rb or control sequence were allowed to migrate for 24 hours. The experiment was performed three times in triplicate. Data are presented as mean ± SD. (C) MCF7ras breast cancer cell line derivatives were analyzed 24 hours after seeding by MTT assay. Data from a representative experiment (n = 5) performed in triplicate are expressed as amount of metabolized MTT measured by absorbance normalized to the absorbance of control shRNA and presented as mean ± SD. (D) Phase contrast images of mammosphere-forming potency of MCF7ras and T47D cells with Rb knockdown. Arrows indicate protrusions formed by invading cells and cell clusters. Scale bar, 50 µm. (TIF) [file pone.0080590.s001.tif]

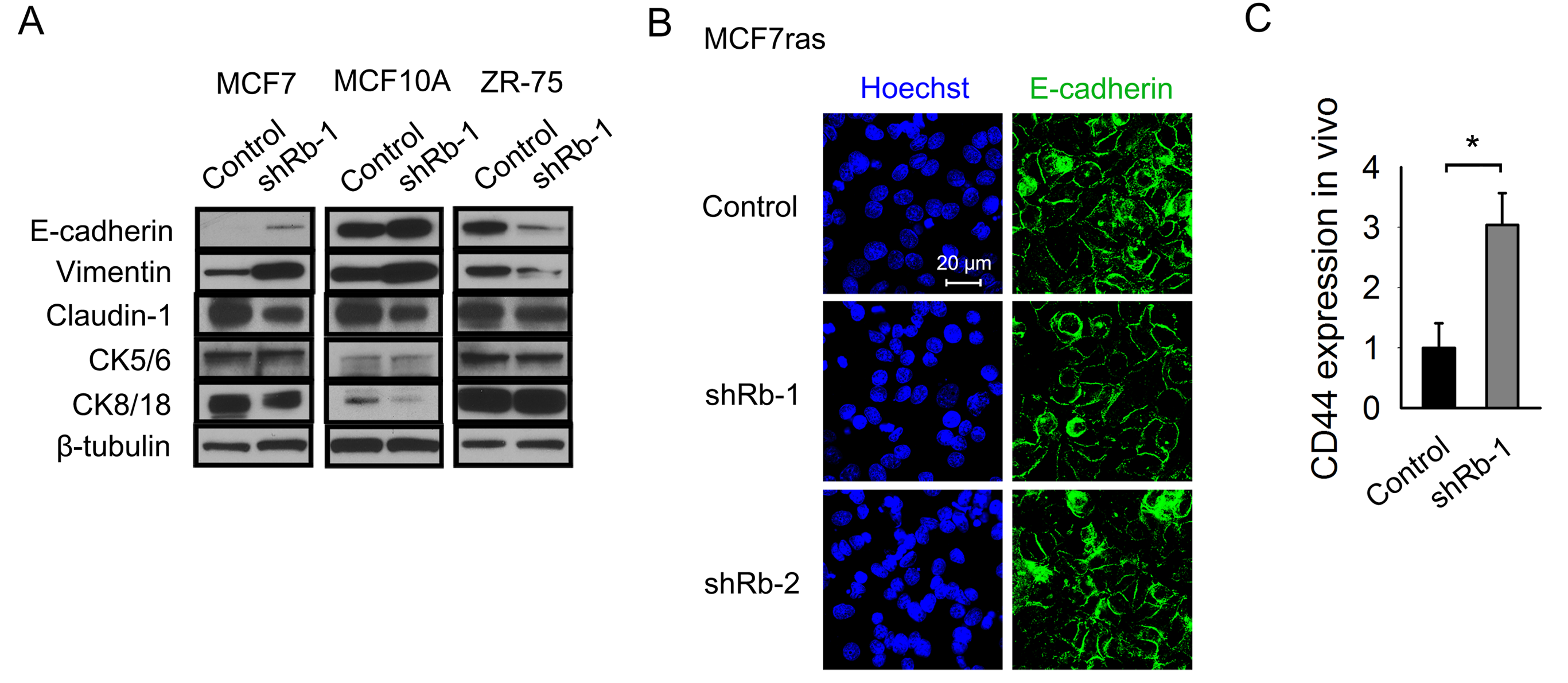

Supplement: Figure S2 — Breast cancer cell lines with Rb knockdown undergo partial EMT. (A) Western blot of cell lysates from MCF7, MCF10A, and ZR-75 cell lines expressing control or Rb shRNA. β-tubulin was used as a loading control. (B) Immunofluorescence image of Rb knockdown MCF7ras cells stained with antibody against E-cadherin (green) and with Hoechst (blue). Scale bar, 20 μm. (C) Quantification of CD44 expression in vivo presented in Figure 2G. Data are depicted as mean ± SD; equal variance Student's t-test, * p<0.05. (TIF) [file pone.0080590.s002.tif]

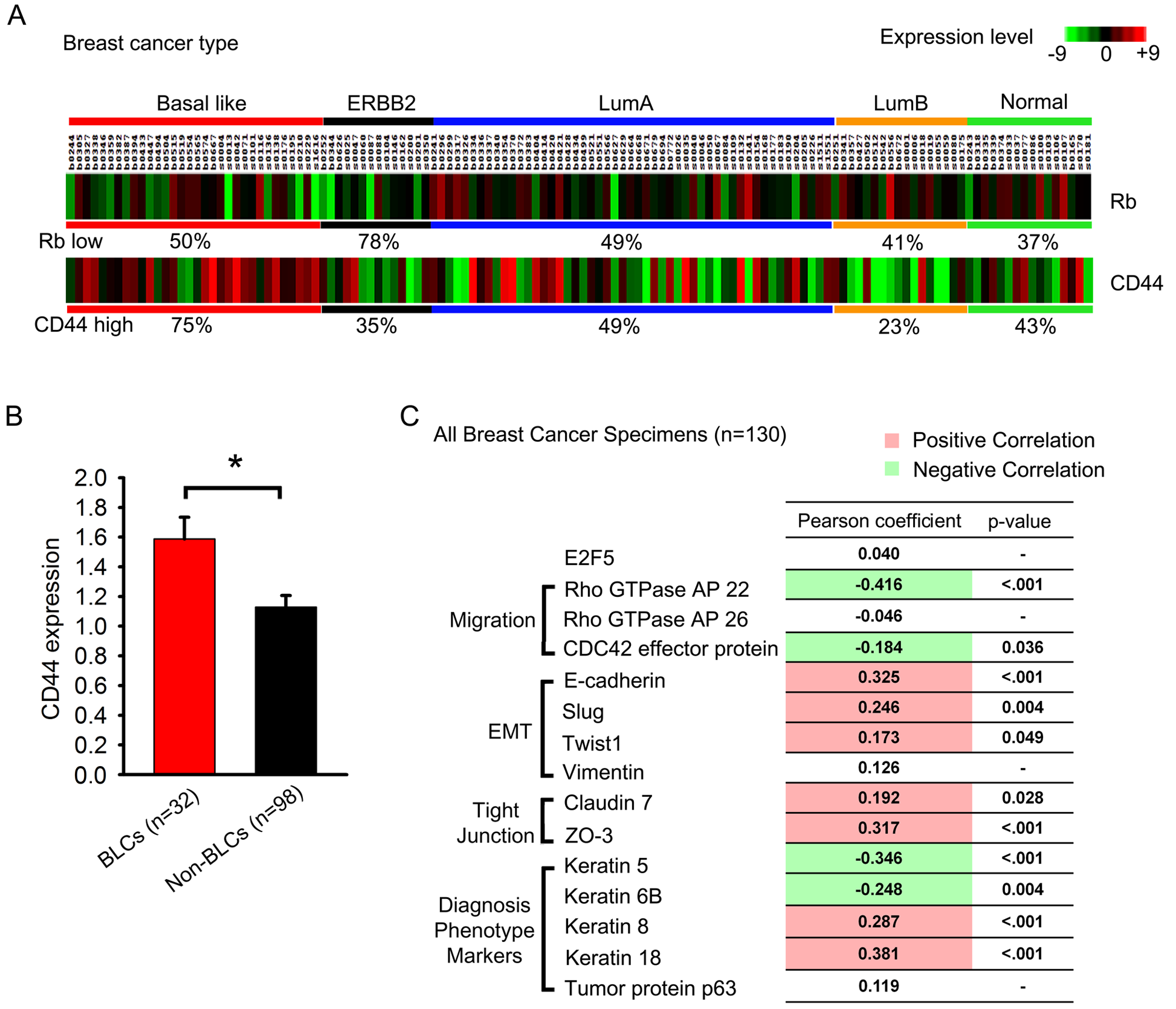

Supplement: Figure S3 — Analysis of mRNA expression data from different types of primary human breast cancers. (A) Comparison of Rb and CD44 mRNA expression in basal-like, ERBB2, luminal A, luminal B, and normal like type of breast cancer. Percentage indicates the fraction of given tumor type featuring Rb-low or CD44-high level. (B) Expression of CD44 mRNA in BLCs versus CD44 mRNA in all other breast cancer specimens. Unequal variance Student's t-test, * p<0.05. (C) Pearson's correlation of Rb expression with CCM related genes, markers of epithelial to mesenchymal transition (EMT), tight junctions, and differentiation across all 130 breast cancer specimens. (TIF) [file pone.0080590.s003.tif]

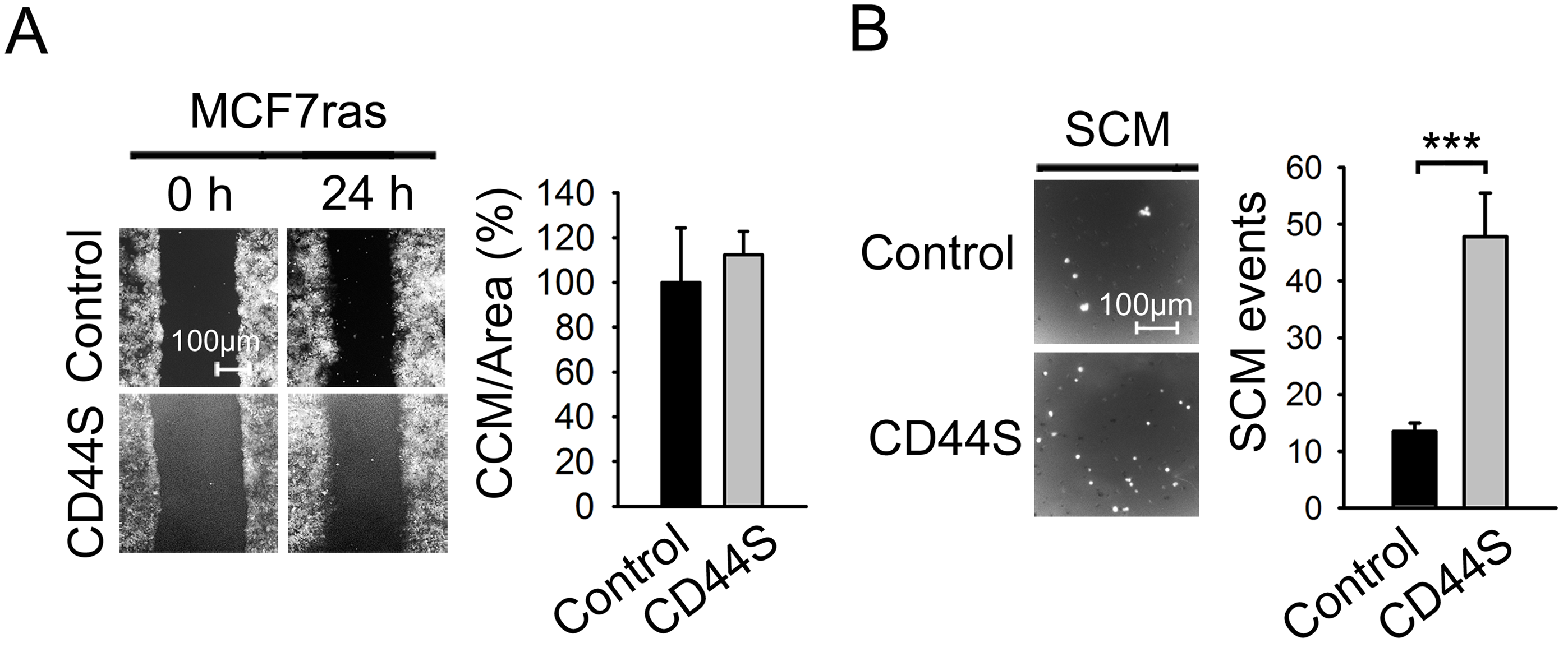

Supplement: Figure S4 — Overexpression of CD44s in Rb positive cells stimulates SCM but not CCM. (A) Quantification of CCM of MCF7ras breast cancer cell line ectopically expressing standard isoform of CD44 or control cDNA. CCM was quantified as an area covered during 24-hour migration, and expressed as a percentage relative to the control. The experiment was performed three times in triplicate. Data are presented as mean ± SD. Scale bar, 100 μm. (B) Quantification of SCM assays. Cells overexpressing CD44 or control cDNA were allowed to migrate for 24 hours. The experiment was performed three times in triplicate. Data are presented as mean ± SD. Scale bar, 100 μm; equal variance Student's t-test, *** p<0.001. (TIF) [file pone.0080590.s004.tif]

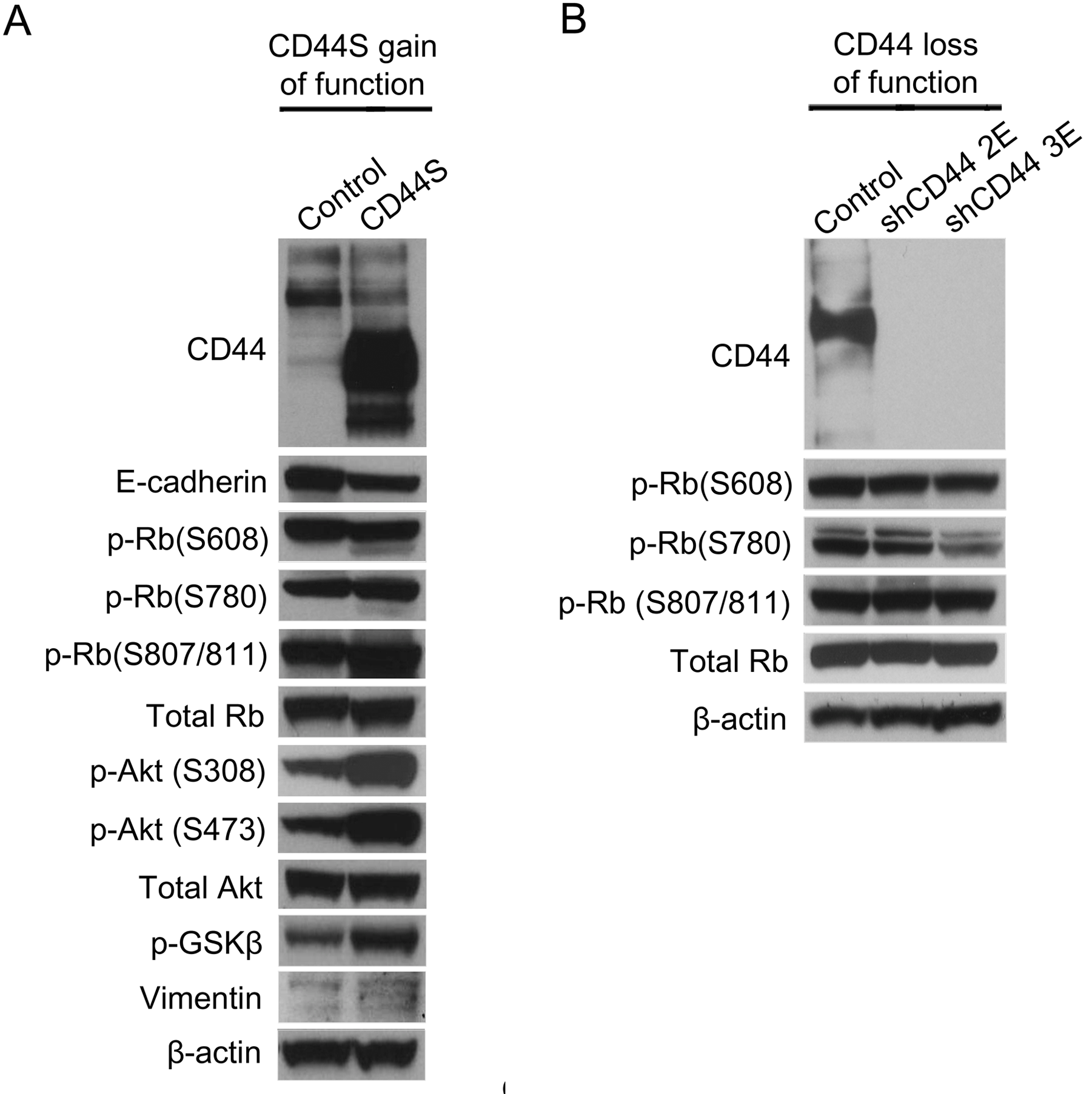

Supplement: Figure S5 — CD44 expression plays a role in Rb phosphorylation. (A) Western blot of cell lysates from MCF7ras cell line ectopically expressing standard isoform of CD44 or control cDNA. β-actin was used as a loading control. (B) Western blot of cell lysates from MCF7ras cell line expressing control or CD44 shRNA. β-actin was used as a loading control. (TIF) [file pone.0080590.s005.tif]

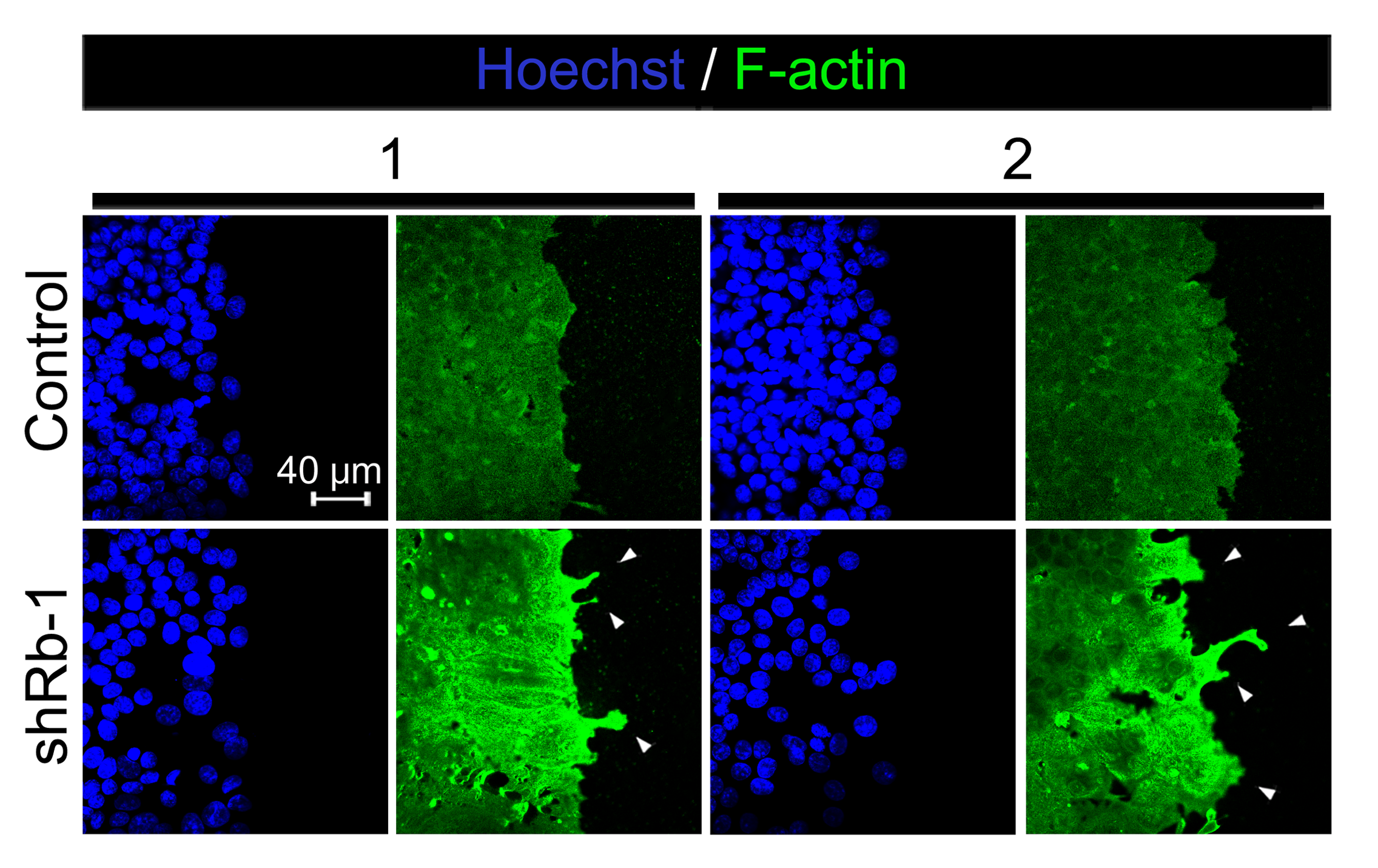

Supplement: Figure S6 — Loss of Rb leads to pronounced extention of F-actin positive filopodia-like formation during CCM. Immunofluorescent analysis of F-actin expression in cells with single Rb knockdowns. Scale bar, 40 μm. (TIF) [file pone.0080590.s006.tif]

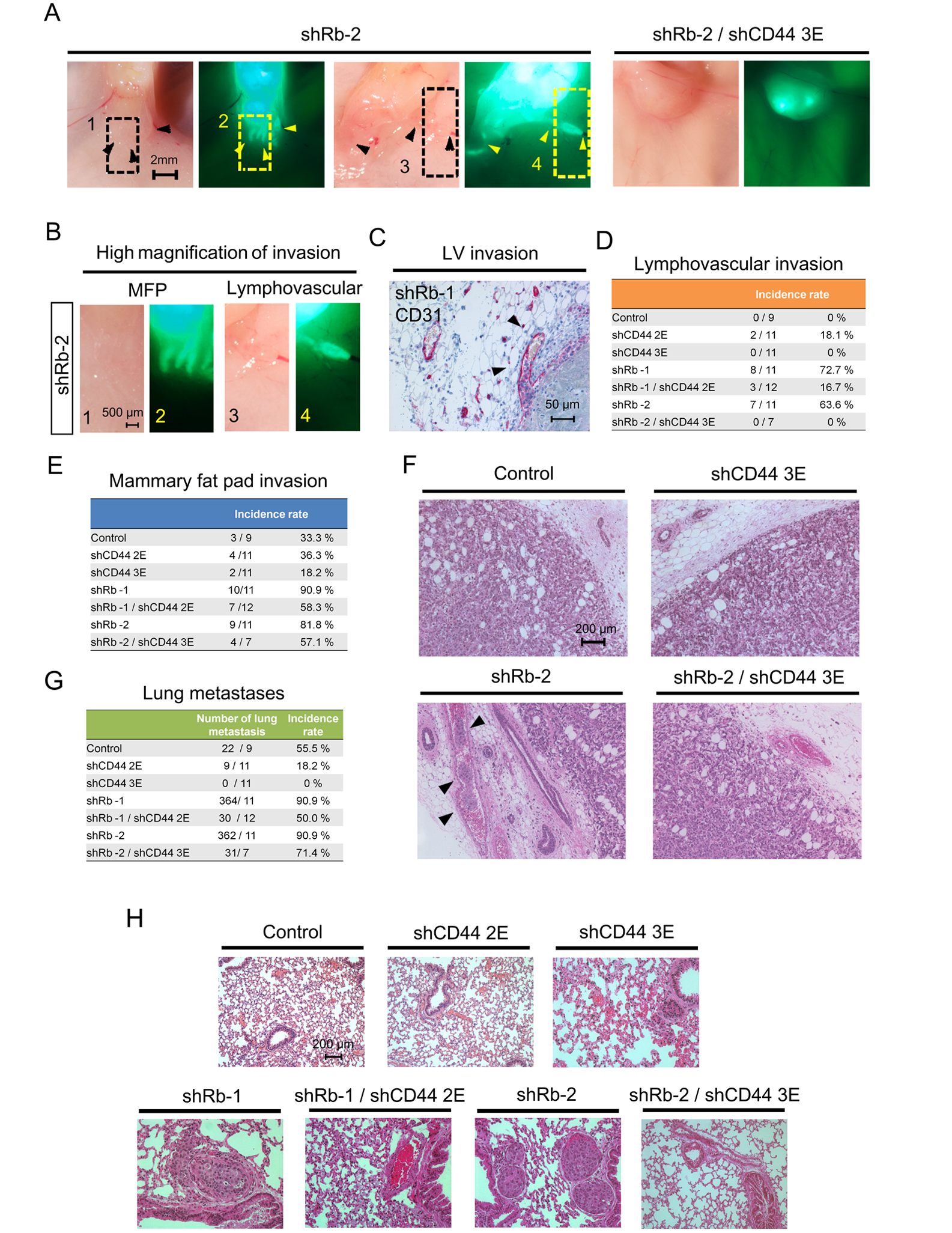

Supplement: Figure S7 — Collective invasion and lung metastases induced by loss of Rb require CD44. (A) Representative phase contrast and fluorescent images of EGFP-positive cancer cells/clusters invading mammary fat pad or adjacent capillaries from orthotopic primary tumor initiated by cells infected with shRNA to Rb and CD44. Analysis was performed on the whole animal post mortem. Scale bar, 2 mm. (B) High magnification phase contrast and fluorescent images of (A). Scale bar, 500 μm. (C) Staining of a sample from primary tumor with anti-CD31 antibody displaying lymphovascular invasion from primary tumor initiated by Rb knockdown cells. Scale bar, 20 μm. (D) Quantification of lymphovascular invasion from primary tumors based on fluorescent images of whole mice. The first number column in the table delineates sum of detected lymphovascular invasion events in the group followed by number of animals in the group. The incidence represents percentage of animals in the group with any detected lymphovascular invasion. (E) Quantification of mammary fat pad invasion from primary tumor based on fluorescent images of whole mice. For this quantification only cell clusters that were considered to be outside of capillary were counted. Sum of all MFP invasion events is followed by number of mice in each group, and percentage of animals in the group with noted event. (F) H&E staining of primary tumors from mice injected with Rb and CD44 double knockdown cells. Arrows indicate areas of lymphovascular invasion as judged by the presence of erythrocytes on the complementary phase contrast images. Scale bar, 200 μm. (G) Quantification of lung metastatic spread from fluorescent images of lungs. Data are expressed as total number of metastases for each group (quantified by ImageJ software) followed by the number of animals in each group. Incidence is the percentage of animals in the group with any detected metastases. (H) H&E staining of lung metastases. Scale bar, 200 μm. (TIF) [file pone.0080590.s007.tif]

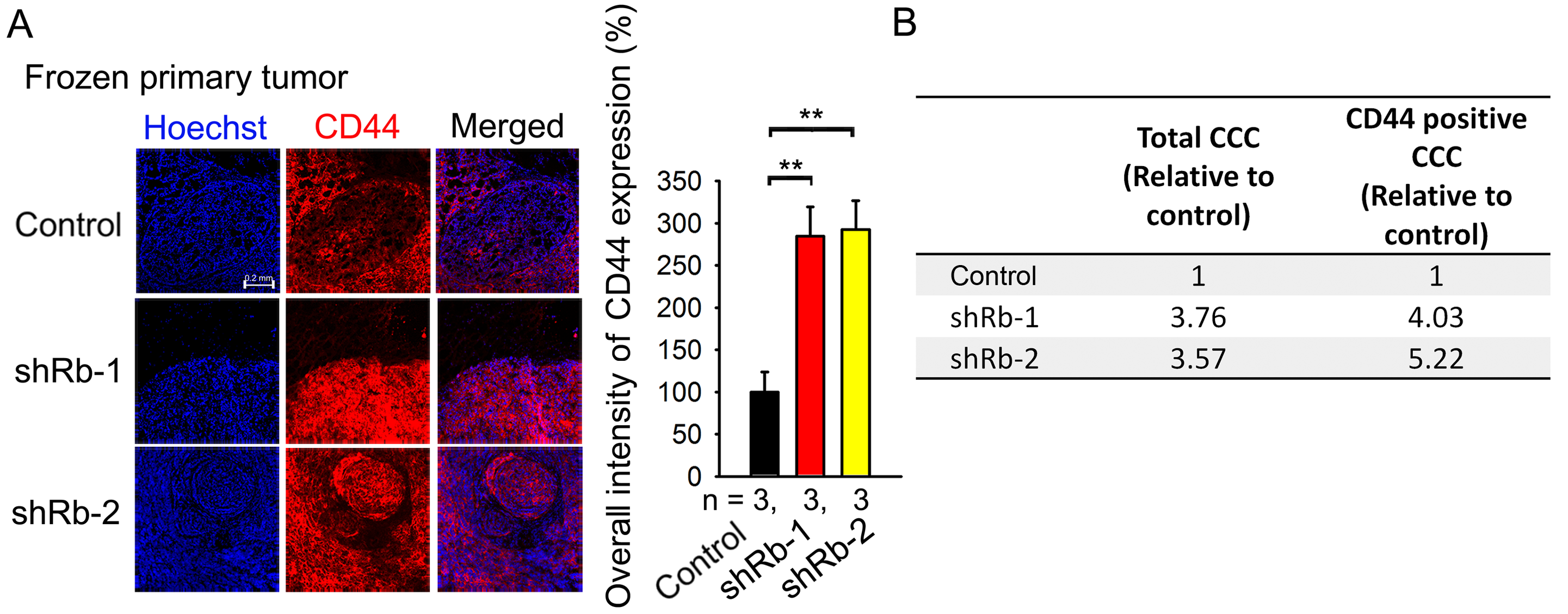

Supplement: Figure S8 — Loss of Rb induces CD44 expression and release of CCC in vivo . (A) Immunofluorescence images and quantification of CD44 expression in Rb knockdown MCF7ras cells stained with antibody against CD44 (red) or Hoechst (blue). Scale bar, 0.2 mm; equal variance Student's t-test, ** p<0.01.(B) Quantification of total CCC and CD44-positive CCC relative to control, related to Figure 6B and 6D. (TIF) [file pone.0080590.s008.tif]
